# Child Life in France

**Published:** 1895-09-14

**Authors:** 


### Social Problems.

### CHILD LIFE IN FRANCE.

The need for special legislation dealing with the interests of children has gradually, of late years, made itself felt in France as in England. The two nations have been working out the problem of the homeless child side by side, and each may gain lessons no less from the failures than from the successes of the other. In one direction, at any rate, England carries off the honour of leading the way for Europe. She was the first nation to grapple courageously with the evils arising out of child labour. M. Bonzon, in his recent able work on the laws relating to childhood,[1] bears generous testimony to the effect produced on Continental opinion by the Factory Acts of 1833 and 1867: "I have dwelt at length on the English laws," he writes, "because they served to initiate our own. Every important step of progress realised in England has been followed sooner or later by a corresponding improvement in France. . . There is now no State of any importance without laws for the regulation of child labour."

In some respects the difficulties of England and France are in sharp contrast. Roughly speaking it may be said that while in France there are too few babies, in England there are too many. Frenchmen are casting envious glances over the Channel at the teeming child population, which taxes all our energies to provide for decently, and as the number of Frenchmen steadily declines, begin to murmur that France is slowly sliding into the abyss. Pessimists in this country who are disposed to lay every trouble at the door of overgrown families, might learn from our neighbours that national prosperity is not necessarily the consequence of that blissful limitation of the family to one boy and one girl in which novelists delight.

There can be no doubt that infant mortality is answerable to a great extent for the scarcity of Frenchmen. A proportion variously estimated at from one-fifth to one-sixth of the total number born is a very large share to spare even from an abundant population. The causes of this excessive rate are complex. But remembering the adverse conditions under which thousands of large slum families are reared in London to be tolerably healthy men and women, it is impossible to resist the conclusion that the difference is to some extent one of racial vigour.

Both nations alike are perplexed by the problem of the foundling. In France it has been the custom for generations, almost to invite mothers to abandon their unwelcome offspring to charity with a view to avoiding all possibility of neglect and loss of life. For this purpose certain "tours" for the secret reception of foundlings were erected by a decree of 1811, in various districts in close proximity to foundling hospitals. "The 'tour' was a cylinder, concave on one side, convex on the other. The concave part, placed on the outside, was destined to receive the abandoned infant; the cylinder revolved to the sound of a bell, and carried the anonymous baby to the sister-in-charge, who assigned it a cradle and a number in the long dormitory of the hospital." About 250 hospitals adopted this device. The results are instructive. The number of foundlings rose from 70,000 in 1810 to 130,000 in 1833. Midwives drove a flourishing trade, sufficient, so the tale runs, to support a large barouche, which collected infants from the outlying districts to drop them in the receptacles conveniently provided in Paris. Moreover, it was found that two-thirds of the infants thus abandoned by their mother died, and the "tours" fell into disuse. French legislation for the illegitimate has now more wisely taken the direction of encouraging the mother by all possible means to keep her child under her own care. Public assistance is granted in France to 40,000 of the class known as *filles-mères*. The mortality among the infants thus retained by their mothers amounts to 25 per cent., which is certainly a high figure, but a wonderful improvement on the 57 per cent. of the infants received into institutions. And it is worth remembering that in old days but 15,000 out of every 100,000 infants received in the Foundling Hospital in Paris survived. Mysterious as it may seem, the poorest, dirtiest, and most ignorant mother is a better guardian of her own baby than can be provided by all the organisation of the best "home" in the world. It is disheartening to find a section of charitable persons in England ready to fall into the very same error which has been so productive of evil in France, by attracting mothers to surrender all responsibility with regard to their illegitimate offspring. The consequences of any widespread scheme of this description must prove ultimately most disastrous to public morality, and, as has been seen, the benefit conferred on the infants is more than doubtful.

The crying evil of France in respect of the child population lies among the beggars. The home supply proving inadequate, an organised system exists of purchasing and hiring children from other countries for begging purposes. It does not appear so far that England has been pressed into the service, notwithstanding her superfluities. But 700 little Italians are annually betrayed into the hands of French agents by their parents, and half of these it is estimated die off in the course of the year. The importation of crippled and infirm children for begging purposes is an even more cruel abuse. According to Dr. Petit, hunchbacks are systematically manufactured at Tolosa, in Spain, whence they are exported into France. "Agents traverse the region on the look out for invalid children. Club feet are in great request. They take the child, at the age of seven or eight years, to the hunchback maker, who, in two months of treatment, undertakes to form a crook, to waste away and dry up the two legs and produce those frightful deformities which are certain to call down a rain of sous." In 1887 the Minister of the Interior, who tried to suppress the infamous traffic, reckoned the number of these hapless children despatched from Spain every year at 400. Again, from Savoy some 1,200 children are annually hired out to beg in the streets of Paris, and all this in addition to a number estimated at 40,000 in the last ten years of true-born Parisian children, some of whom start life as babies, hired out to excite compassion, and fetching their highest price by night in bad weather. When will a Parisian counterpart of Dr. Waugh arise to conquer?

[1] La Legislation de l'Enfance, par Jacques Bonzon, Avocat à la Cour d'Appel. Paris: Libraire Guillamin and Co.

